# Xylanase and Fermented Polysaccharide of *Hericium caputmedusae* Reduce Pathogenic Infection of Broilers by Improving Antioxidant and Anti-Inflammatory Properties

**DOI:** 10.1155/2018/4296985

**Published:** 2018-12-30

**Authors:** Sitong Zhang, Chunfeng Wang, Yang Sun, Gang Wang, Huan Chen, Dan Li, Xiaoxiao Yu, Guang Chen

**Affiliations:** ^1^College of Life Science, Jilin Agricultural University, Changchun 130118, China; ^2^College of Animal Science and Technology, Jilin Agricultural University, Changchun 130118, China; ^3^College of Agriculture, Jilin Agricultural University, Changchun 130118, China

## Abstract

**Background:**

Pathogenic infection in broilers has become an important issue in the development of poultry industry. Xylooligosaccharides released from xylan via xylanase and fermented polysaccharide of *Hericium caputmedusae* (FPHC) have antimicrobial potential against many pathogens.

**Objective:**

We aimed to explore the effects of xylanase and FPHC on pathogenic infection in the broilers (*Gallus gallus domesticus*).

**Methods:**

Three hundred and thirty 21-day male broilers were assigned into four groups: control group (CG, basic diet), xylanase group (XG, basic diet + xylanase), FPHC group (HG, basic diet + FPHC), and XHG group (basic diet + xylanase + FPHC). Average daily feed intake (ADFI) and daily gain (ADG) were measured. Microflora from broiler feces was analyzed using 16S rRNA sequencing. Serum tumor necrosis factor- (TNF-) *α*, interleukin-1*β* (IL-1*β*), IL-1 receptor antagonist (IL-1ra), IL-10, total antioxidant capacity (T-AOC), superoxide dismutase (SOD), glutathione peroxidase (GSH-Px), and malondialdehyde (MDA) contents were detected using kits. The variables were compared using the Student *t*-test between two groups.

**Results:**

Microbiological investigations showed that 75% of broilers were affected by bacterial pathogens in the CG group, most notably by coagulase-negative staphylococci. Comparatively, 15%, 26%, and 5% of broilers were affected by bacterial pathogens in the XG, HG, and XHG groups, respectively. Xylanase and FPHC treatment increased the ratio of ADG to ADFI and antioxidant capacity by increasing the levels of T-AOC, SOD, and GSH-Px and reducing the levels of MDA (*P* < 0.05). Xylanase and FPHC treatment improved anti-inflammatory capacity by increasing serum levels of IL-1ra and IL-10 and reducing the levels of IL-1*β* and TNF-*α*. On the other hand, the treatment increased probiotic concentration of *Bacillus licheniformis*, *Bacillus subtilis*, and *Lactobacillus plantarum* (*P* < 0.05), which were also proved in cell culture.

**Conclusions:**

Xylanase and FPHC ameliorate pathogen infection by increasing antioxidant and anti-inflammatory activities of broilers via the increase of probiotics.

## 1. Introduction

Avian pathogens have become an important issue in the development of poultry industry. Antibiotic is often considered and immeasurable in poultry industry. However, antibiotic-resistant pathogens have become a public health issue and affect the composition of microbiota in poultry [1] and poultry production [2, 3]. It is highly demanded to explore antibiotic alternatives in poultry industry [4].

As emerging prebiotics, the production of xylooligosaccharide from biomass by xylanases was also widely reported [5–7]. The xylooligosaccharides, released from xylan via xylanase, have been proved to exhibit beneficial commensals by decreasing pathogenic bacteria and increasing bifidobacteria [5]. Xylooligosaccharides had antibacterial potential against many pathogens, including *Klebsiella pneumoniae*, *Enterococcus faecalis* [8], and *Helicobacter pylori* [9]. On the other hand, xylooligosaccharide has been reported to maintain gut flora balance by promoting the growth of probiotics, such as *Lactobacillus* spp. and *Bifidobacterium* spp., and eliminating enteric pathogens, such as *Clostridium perfringens* [10]. Polysaccharide from *Hericium caputmedusae* was also reported to improve gut microflora [11].

On the other hand, the composition of intestinal probiotics will affect broiler immune [12], intestinal microarchitecture [13], and microbial profiles [14]. Furthermore, probiotics have antioxidant [15] and anti-inflammatory [16] properties. Oligosaccharide can improve probiotic effect of intestinal flora [17], and probiotics can use xylooligosaccharides produced from xylan by xylanase [18].

Furthermore, xylooligosaccharide in diets can enhance the growth rate, modulate endocrine metabolism, and improve immune function in poultry [19]. Nonstarch polysaccharide was also found to improve the growth performance of poultry [20]. More work also showed that supplementing pelleted diets with thermoresistant multienzyme improved broiler performance [21]. However, little data are available for the effects of polysaccharide from *H. caputmedusae* and xylanase on pathogenic infection in broilers. Poultry infection has been found to be associated with antioxidant and anti-inflammatory activities. Therefore, the effects of xylanase and polysaccharides from *Hericium caputmedusae* on pathogenic infection in broiler were explored by investigating antioxidant and anti-inflammatory activities and changes of intestinal microbiota.

## 2. Materials and Methods

### 2.1. Broilers and Diets

Before the experiment, all procedures were approved by the Animal Research Committee of Jilin Agricultural University (approval no. 20150123JAUA1, Changchun, China). *H. caputmedusae* was purchased from Jilin University Pharmaceutical Factory (Changchun, China). Xylanase was purchased from Hunan New Century Biochemical Co. Ltd. (Yueyang, China). Three hundred and thirty 21-day male broilers (*Gallus gallus domesticus*) (0.9–1.0 kg) were purchased from Changchun Yongxu Animal Husbandry and Veterinary Company (Changchun, China), and randomly and evenly assigned into four groups according to different treatments (Table 1): control group (CG, the broilers received basic diet), xylanase group (XG, the broilers received basic diet and xylanase with activity of 1200 IU/kg), polysaccharide group (HG, the broilers received basic diet and 0.1% fermented polysaccharides *H. caputmedusae* (FPHC, *w*/*w*)), and xylanase combined with FPHC group (XHG, the broilers received basic diet, 1200 IU/kg of xylanase, and 0.1% FPHC (*w*/*w*)). Each of the groups was assigned into three subgroups with 30 broilers in a room (250 cm × 230 cm × 250 cm, length × width × height) and given ad libitum access to feed and water. Each room was controlled at 20°C. The basal diets were fed and prepared in a feed mill. A basal diet was formulated to meet the requirements by National Research Council (NRC) (Table 1, in the CG group) [22]. All broilers were reared in natural light and dark. Average daily gain, mortality, average daily feed intake (ADF), and feed-to-gain ratio were measured.

### 2.2. *H. caputmedusae* Culture

*H. caputmedusae* was transferred from a slant medium to a PDA medium and cultured in a 24°C incubator. After one-week culture, one cm^2^ of medium with mycelia was transferred to 400 mL of a liquid medium and cultured in a shaker for 10 d at 140 rpm and 25°C. After 30-day fermentation, the mycelium was separated from the fermentation broth and dried. The mycelium was broken using an ultrasonic device, and polysaccharides were isolated by adding distilled water and placed at room temperature for 2 h. The mixture was centrifuged at 12,000*g* for 10 min, and the supernatants were collected. The steps were repeated for 3 times.

### 2.3. Preparation of Polysaccharides from *H. caputmedusae*

Three-fold volume of 95% ethanol was added to the fermentation broth and placed at room temperature for 36 h. Precipitates were collected via centrifugation at 12,000*g* for 30 min. Protein was removed by adding the mixed solution (n-butanol : chloroform volume, 1 : 4) at 1 : 10 *w*/*v*. Finally, purified polysaccharide was obtained.

### 2.4. Sample Collection

On the days 21 and 42, eight broilers in each group were slaughtered by severing the jugular veins. Small intestinal contents were harvested immediately and transported to the laboratory for counting microbial colonies. Intestinal mucosa was nipped by forceps and rinsed in 0.85% saline solution. Mucosa was scraped by blunt side of surgical knife blades, collected in microtubes immediately, frozen in liquid nitrogen, and stored at −80°C until the next steps.

### 2.5. Biochemical Analysis

Approximately two-milliliter blood was taken from per broiler, and serum was prepared via centrifugation at 1500 rpm for 10 min. The levels of superoxide dismutase (T-SOD) [23], total antioxidant capacity (T-AOC) [24], glutathione peroxidase (GSH-Px) [25], and malondialdehyde (MDA) [26] have been reported as the biomarkers of oxidative stress. Therefore, the levels of all these molecules were measured in serum samples using the kits from Beyotime Institute of Biotechnology (Jiangsu, China). Inflammation is closely associated with the changes of the distribution of intestinal flora. Therefore, inflammatory situation was detected by measuring serum (tumor necrosis factor) TNF-*α*, (interleukin) IL-1*β*, IL-1 receptor antagonist (IL-1ra), and IL-10 via chicken ELISA Kit from Cusabio (College Park, MD, USA).

### 2.6. Amplification of 16S rRNA

Ten-milligram feces were collected from intestine of each broiler and diluted in water by 100-fold. Genomic DNA was extracted from the sample using a DNA Isolation Kit (Promega, Madison, WI, USA). DNA samples were analyzed using an ND-2000 spectrophotometer (NanoDrop Inc., Wilmington, DE, USA).

Isolated DNA was used as a template to amplify 16S rRNA gene regions using universal primers: forward primer, 5′-AGRGTTYGATYMTGGCTCAG-3′, and reverse primer, 5′-TTACCGCGGCTGCTGGCAC-3′. PCR mixture consisted of 39 *μ*L ddH_2_O, 1 *μ*L DNA genome, 5 *μ*L 10x buffer, 0.5 *μ*L Taq DNA polymerase (Takara, Dalian, China), 0.5 *μ*L forward and reverse primers (20 *μ*M), and 4 *μ*L dNTP, in total of 50 *μ*L. PCR was performed with the following condition: 95°C for 5 min, 30 cycles of 95°C sec for 20 sec, 55°C for 30 sec, 72°C for 1 min 30 sec, and 72°C for 5 min for final extension. PCR products were determined on 1% agarose gel and gel-purified.

### 2.7. Microbiota Analysis

Sample for bead-based sequencing was set up according to an earlier report [27] and sequenced using Roche 454 GS FLX platform on GS FLX instruments from Roche (Roche, Nutley, NJ, USA) [28]. The heat map of 16S rRNA gene sequences on the 21st day and 42nd day was created using Genomics Viewer at http://www.broadinstitute.org/igv. The R packages stats were used to perform statistical analysis. Simpson index and Shannon index were used to analyze the community diversity among different groups [29].

### 2.8. Microorganism

*Bacillus licheniformis*, *Bacillus subtilis*, and *Lactobacillus plantarum* were purchased from Institute of Microbiology, Chinese Academy of Sciences (Beijing, China). The seed was transferred to 100 mL of a basal medium with glucose 10.0 g/L, peptone 10.0 g/L, K_2_HPO_4_ 1.0 g/L, MgSO_4_ 0.2 g/L, and Na_2_CO_3_ 5.0 g/L (pH 7.0) in a 250 mL Erlenmeyer flask. 1200 IU/kg of xylanase or 0.1% FPHC was added to the medium. The strains were cultured in a thermostatic orbital shaker for 48 h, at 37°C and 200 rpm. Samples were withdrawn at regular intervals, and probiotics were counted by serial dilution of the material in sterile distilled water and plating on a LB agar plate. The bacterial numbers were counted by observing the colony on the plate after one-day culture at 37°C.

### 2.9. Statistical Analysis

All data were presented as mean value ± S.D. The Student *t*-test was used to compare the variables between two groups using SPSS software version 20.0 (SPSS Inc., Chicago, IL, USA). There is a significant difference if *P* < 0.05.

## 3. Results

### 3.1. Growth Performance

The statistical difference of the ratio of feed to gain was insignificant among four groups before the 21st day (Table 2, *P* > 0.05). Compared with CG, Table 2 showed that the broilers had a higher ratio of feed to gain when they received xylanase or FPHC (*P* < 0.05). The ratio was highest when the broilers received both xylanase and FPHC (*P* < 0.05). No mortality was observed during the whole experimental period.

### 3.2. Xylanase and FPHC Treatment Increases the Numbers of Intestinal Bacterial Species of Broilers

**Figure 1:**
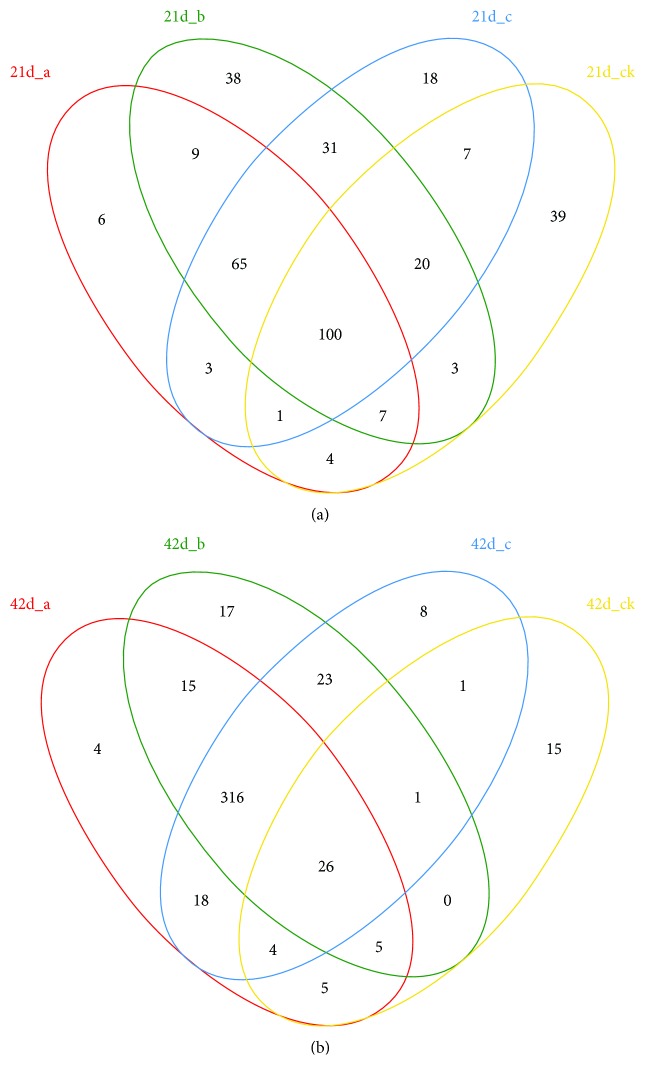
The effects of xylanase and FPHC treatment on the numbers of intestinal bacterial species of broilers. (a) The numbers of bacterial species of broilers among four groups before the 21st day. (b) The numbers of bacterial species of broilers among four groups on the 42nd day.

The numbers of bacterial species of broilers were similar among four groups before the 21st day (Figure 1(a), *P* > 0.05). Xylanase and FPHC treatment increased the numbers of bacterial species of broilers while the number was reduced significantly in the control group on the 42nd day when compared with other groups (Figure 1(b), *P* < 0.05).

### 3.3. Xylanase and FPHC Treatment Increases the Concentration of Probiotics

Heat map analysis showed that xylanase and FPHC treatment increased the concentration of probiotics, including *Lactobacillus* and *Bacillus* species. For other species, *Anaerotruncus*, *Candidatus* Arthromitus, *Pseudomonas*, *Lachnospiraceae*, *Enterococcus*, *Stenotrophomonas*, and *Acinetobacter* were increased in the control group while *Lactococcus*, *Blautia*, *Subdoligranulum*, *Flavonifractor*, and *Lachnoclostridium* were increased in xylanase and FPHC groups.

The concentration of *L. plantarum* [30], *B. licheniformis* [31], and *B. subtilis* [32] was measured in the intestine of broilers and compared on the 21st day and 42nd day using feces among four groups (Table 3). The statistical difference for the concentration was insignificant among four groups before the 21st day (Table 3, *P* > 0.05). Compared with the broilers in the control group, the concentration of these probiotics was higher in the xylanase or FPHC group on the 42nd day (*P* < 0.05). These results suggest that xylanase and FPHC treatment increases the concentration of probiotics.

### 3.4. Xylanase and FPHC Treatment Increases Antioxidant Activities of Broilers

**Figure 2:**
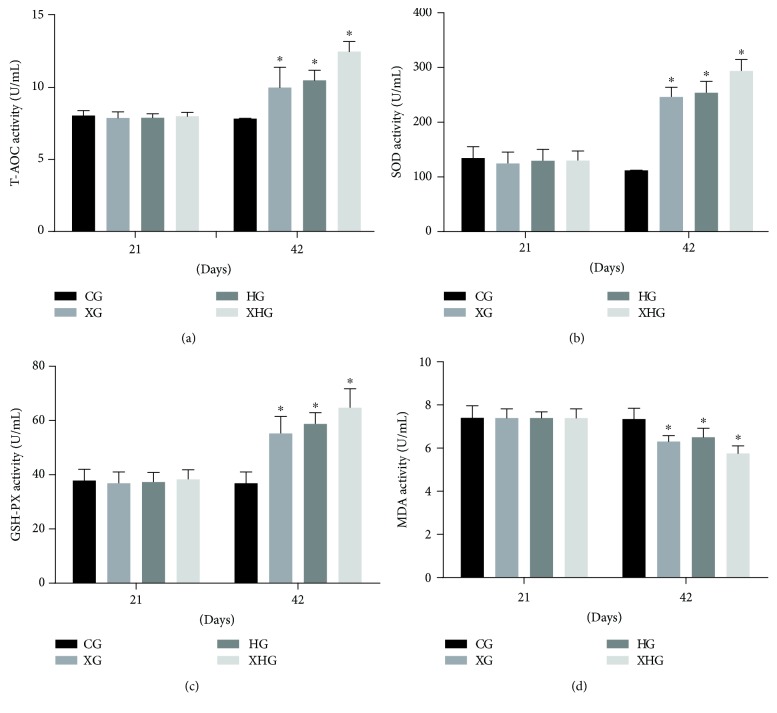
The effects of xylanase and FPHC on antioxidant activities of broilers. (a) The effects of xylanase and FPHC on T-AOC activities of broilers. (b) The effects of xylanase and FPHC on SOD activities of broilers. (c) The effects of xylanase and FPHC on GSH-PX activities of broilers. (d) The effects of xylanase and FPHC on MDA activities of broilers.

The antioxidant properties were measured by investigating the activities of T-AOC, SOD, GSH-px, and MDA in the intestine of broilers among four groups. Before xylanase and FPHC treatment, there was no significant difference for antioxidant activities among four groups (Figure 2, *P* > 0.05). Compared with the broilers in the CG group, the activities of T-AOC (Figure 2(a)), SOD (Figure 2(b)), and GSH-PX (Figure 2(c)) were increased in XG, HG, and XHG groups while the activity of MDA (Figure 2(d)) was reduced in XG, HG, and XHG groups after xylanase and FPHC treatment (*P* < 0.05). These results suggest that xylanase and FPHC treatment increases the antioxidant activities of broilers.

### 3.5. Xylanase and FPHC Treatment Increases Anti-Inflammatory Activities of Broilers

**Figure 3:**
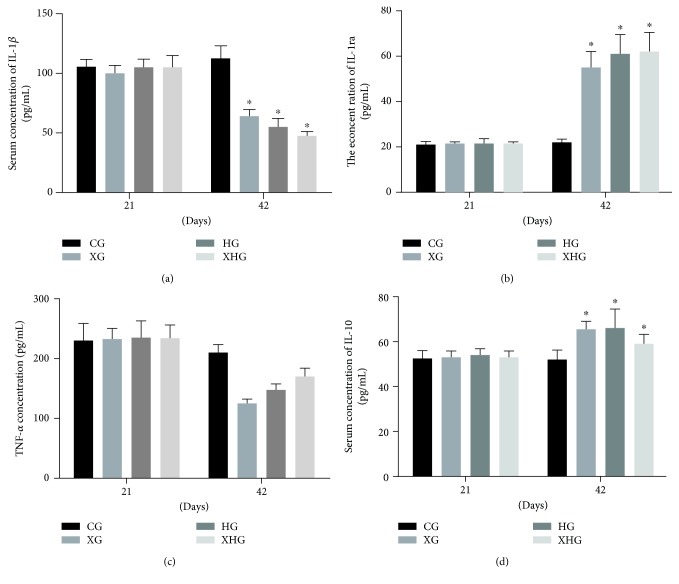
The effects of xylanase and FPHC on anti-inflammatory activities of broilers. (a) The effects of xylanase and FPHC on the concentration of IL-1*β* of broilers. (b) The effects of xylanase and FPHC on the concentration of IL-1ra of broilers. (c) The effects of xylanase and FPHC on the concentration of TNF-*α* of broilers. (d) The effects of xylanase and FPHC on the concentration of IL-10 of broilers.

The anti-inflammatory properties were measured by investigating the serum concentrations of IL-1*β*, IL-1ra, TNF-*α*, and IL-10 in the intestine of broilers among four groups. Before xylanase and FPHC treatment, there was no significant difference for the concentrations of the cytokines among four groups (Figure 3, *P* > 0.05). Compared with the broilers in the control group, the concentrations of IL-1ra (Figure 3(b)) and IL-10 (Figure 3(d)) were increased in XG, HG, and XHG groups while the concentrations of IL-1*β* (Figure 3(a)) and TNF-*α* (Figure 3(c)) were reduced in the three groups after xylanase and FPHC treatment (*P* < 0.05). These results suggest that xylanase and FPHC treatment increases the anti-inflammatory activities of broilers.

### 3.6. Xylanase and Polysaccharide Reduce Pathogen Infection Rates of Broilers

Microbiological investigations demonstrated that 75% of broilers were affected by bacterial pathogens in the CG group, most notably by coagulase-negative staphylococci (Table 4). Comparatively, 15%, 26%, and 5% of broilers were affected by bacterial pathogens in the XG, HG, and XHG groups, respectively. These findings suggest that coagulase-negative staphylococci are prevalent in the local area. FPHC and xylanase can control and prevent bacterial pathogen prevalence.

### 3.7. Xylanase and FPHC Treatment Promoted the Growth of Probiotics

**Figure 4:**
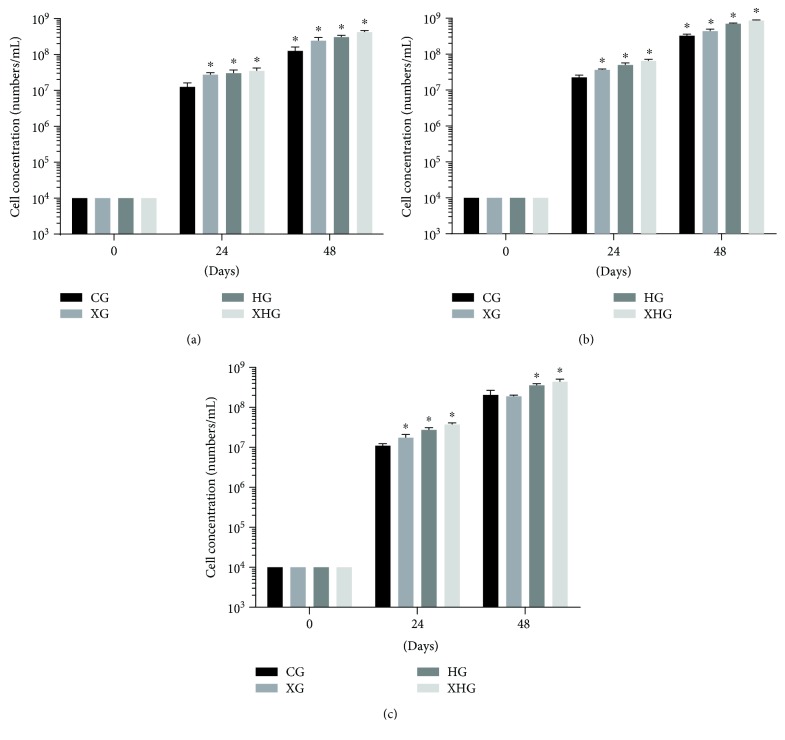
The effects of xylanase and FPHC treatment on the growth of probiotics. (a) The effects of xylanase and FPHC treatment on the growth of *Bacillus licheniformis*. (b) The effects of xylanase and FPHC treatment on the growth of *Bacillus subtilis*. (c) The effects of xylanase and FPHC treatment on the growth of *Lactobacillus plantarum*. ^∗^*P* < 0.05 vs. a control group.

The growth-promoting properties of xylanase and FPHC were measured using *B. licheniformis*, *B. subtilis*, and *L. plantarum* via cell culture. At 0-hour culture, there was no significant difference for cell concentration among three species (Figure 4, *P* > 0.05). At 24-hour culture, xylanase and FPHC treatment increased the cell concentrations of *B. licheniformis* (Figure 4(a)), *B. subtilis* (Figure 4(b)), and *L. plantarum* (Figure 4(c)) when compared with the controls (*P* < 0.05). At 48-hour culture, xylanase and FPHC treatment also further increased more cell concentrations than controls (Figure 4, *P* < 0.05). These results suggested that xylanase and FPHC treatment promoted the growth of these probiotics.

## 4. Discussion

Xylanase and FPHC have a beneficial effect on the physiology, health, and productivity of broilers. Early studies demonstrated that xylanase resulted in higher weight gain in broilers when compared to controls without xylanase addition [33]. The diets with xylanase will affect the animal growth rate by improving the utilization of nutrient [34]. Xylanase prolongs the retention time of fiber in the intestinal tract, and more nutrient can be absorbed. Furthermore, longer duration of fiber in the intestinal tract will result in better microbial adaptation [35].

It has been well known that the intestinal microbiota is an important determinant for gastrointestinal health of broilers. Probiotics have the potential to improve the beneficial bacteria and inhibit pathogenic bacteria. Supplementation of prebiotic will eliminate pathogenic bacteria and increase probiotics, which have beneficial effects on broiler growth and immune-related gene expression [12]. Probiotics also provide protection against bacterial infection [36]. *B. subtilis* supplementation in diet will affect the diversity, composition, and functional diversity of the fecal microbiota in broiler [37]. *B. licheniformis* improves the growth and antioxidant abilities of broilers. Meanwhile, the probiotics can affect the expression of genes associated with fatty acid synthesis and oxidation [38]. *L. plantarum* can effectively replace in-feed antibiotic and improve the intestinal health by changing intestinal villus morphology and inhibiting the pathogenic loading [39]. *Lactobacillus* species effectively absorb and expel heavy metal toxicity from the gastrointestinal tract of broilers [40]. The present study showed that xylanase and FPHC treatment increased the concentrations of *L. plantarum*, *B. licheniformis*, and *B. subtilis* in the small intestine (Table 3). *B. licheniformis* and *B. subtilis* are aerobes and use oxygen in the intestine, resulting in an oxygen-free environment for the proliferation of anaerobic probiotics such as *Lactobacillus*. The probiotics will produce more acidic environments, which control the growth of potential pathogens.

Most diseases of broilers are associated with oxidative damage [41]. The studies focused on antioxidant molecules in broilers. Dietary antioxidants can minimize the negative effect of oxidized oil on meat qualities of broilers [42]. Our results showed that xylanase and FPHC treatment increased the level of T-AOC, SOD, and GSH-PX and reduced the level of MDA (Figure 2). These changes had beneficial effects on broilers by improving their antioxidant activities.

Reducing enteric inflammation and maintaining intestinal homeostasis are very important to improve the growth of broilers. Probiotics can improve immunomodulatory activity and are effective in controlling *Salmonella* colonization, invasion, and the induced inflammation [43]. The levels of lymphocyte phenotypes (including B and T lymphocytes) and plasma immunoglobulin in broilers are also associated with their infected diseases [44]. Present findings demonstrated that xylanase and FPHC treatment increased the anti-inflammatory activities of broilers by increasing the levels of IL-1ra and IL-10 and reducing the levels of IL-1*β* and TNF-*α* (Figure 3). Furthermore, cell culture showed that xylanase and FPHC treatment also promoted the growth of probiotics (Figure 4).

T-AOC is the antioxidant capacity of the body's defense system and can fully reflect the antioxidant capacity of both enzyme and nonenzymatic systems. Although it does not clearly represent the activity of an antioxidant or antioxidant enzyme, it reflects antioxidant capacity better than a single indicator. SOD, one of the members of the enzymatic system, specifically and efficiently scavenges superoxide radicals. It is the only enzyme known to directly eliminate O^2−^ and protect cells. This study showed that xylanase + FPHC treatment increased serum T-AOC levels, SOD, and GSH-PX activity and reduced the serum MDA level. MDA is one of the most toxic lipid peroxides, and it can not only destroy the membrane structure and membrane protein function and affect the function and metabolism of nucleic acids but also cause autoimmune disorders. Therefore, the determination of MDA can reflect the degree of lipid peroxidation and help to understand damage degree of tissue and cells. Xylanase and FPHC not only have a wide range of activity and diversity but also have abundant sources, low costs, and good safety. They have a good application prospect in animal husbandry production.

There are some limitations of the present work: (1) the changes for tissue morphology and intestinal barriers were not investigated here. The work only reflected the changes for inflammatory and infection situations in broilers; (2) the functions of most species of the microbiota in broilers were not analyzed in the present study; (3) the effects of xylanase and HPFC on antioxidant and anti-inflammatory signaling pathway were not explored either. Thus, further work is still needed to perfect present results in the future.

## 5. Conclusions

Xylanase and FPHC can effectively increase the serum T-AOC, SOD, and GSH-PX activity and reduce the MDA content to improve the broiler's antioxidant activities. Xylanase and FPHC treatment also maintained intestinal species in a healthy situation. Meanwhile, the addition of xylanase and FPHC in diet increased broiler's anti-inflammatory capacity by increasing the levels of IL-1ra and IL-10 and reducing the levels of IL-1*β* and TNF-*α*. Broilers were affected by bacterial pathogens, most notably by coagulase-negative staphylococci. Xylanase and FPCH treatment ameliorated pathogen infection of broilers by increasing the amounts of probiotics *B. licheniformis*, *B. subtilis*, and *L. plantarum*. For poultry, because of its special digestive tract structure characteristics, the role of the antioxidant effect of xylanase and FPHC may be different and remains to be studied.

## Figures and Tables

**Table 1:** Ingredients and chemical composition of diets among different groups.

Ingredients (g/kg)	CG	XG	HG	XHG
Corn	595.0	595.0	595.0	595.0
Soybean meal (47% CP)	330.0	330.0	330.0	330.0
Corn oil	35.0	35.0	35.0	35.0
CaHPO_4_·2H_2_O	13.0	13.0	13.0	13.0
Limestone	13.0	13.0	13.0	13.0
Xylanase (IU/kg)^∗^	0	1200	0	1200
FPHC^∗^	0	0	1	1
Salt	3.0	3.0	3.0	3.0
DL-Met	1.0	1.0	1.0	1.0
Premix^a^	10.0	10.0	10.0	10.0
Chemical analysis (g/kg)				
CP	209.9	209.8	209.8	209.9
Crude fiber	23.2	23.3	23.2	23.3
Ether extract	55.2	55.3	55.2	55.2
Crude ash	62.3	62.5	62.4	62.4
Ca	8.8	8.9	8.9	8.8
*P*	3.5	3.6	3.6	3.6
Calculated analysis				
ME (MJ/kg)	13.3	13.3	13.3	13.3
Lys (%)	1.10	1.10	1.10	1.10
Thr (%)	0.78	0.78	0.78	0.78
TSAA (%)	0.83	0.83	0.83	0.83

Note: xylanase was added at 1200 IU/kg. FPHC: fermented polysaccharides of *Hericium caputmedusae*. ^a^Premix provided the following per kilogram of diet: vitamin A (retinyl palmitate), 9000 IU; vitamin D3, 2000 IU; vitamin E (DL-*α*-tocopheryl acetate), 10.0 mg; vitamin K, 0.5 mg; vitamin B1, 1.8 mg; vitamin B6, 3.5 mg; vitamin B12, 0.01 mg; riboflavin, 3.6 mg; niacin, 35.0 mg; pantothenic acid, 10.0 mg; folic acid, 0.55 mg; biotin, 0.15 mg; choline chloride, 250 mg; Mn, 60.0 mg; Zn, 40.0 mg; Fe, 80.0 mg; Cu, 8.0 mg; I, 0.35 mg; and Se, 0.15 mg. CG: the broiler received basic diet; XG: the broiler received basic diet and 1200 IU/kg xylanase (Hunan New Century Biochemical Co. Ltd., Yueyang, China); HG: the broiler received basic diet and 0.1% polysaccharides from the fermentation extract of *Hericium caputmedusae* (FPHC, *w*/*w*); XHG: the broiler received basic diet, 1200 IU/kg of xylanase, and 0.1% FPHC (*w*/*w*). ^∗^*P* < 0.05 among four groups.

**Table 2:** Effects of xylanase and FPHC on growth performance of broilers (*n* = 8).

Groups		ADG (g)	ADFI (g)	Feed/gain
CG	21 d	35.8 ± 3.6	72 ± 8	1.85 ± 0.16
	42 d	71.0 ± 6.5	151 ± 13	2.13 ± 0.18^b,c,d^

XG	21 d	35.1 ± 3.3	70 ± 9	1.79 ± 0.15
	42 d	76.8 ± 6.1	148 ± 15	1.93 ± 0.17^a,d^

HG	21 d	36.1 ± 3.4	73 ± 7	1.90 ± 0.18
	42 d	77.5 ± 7.3	146 ± 12	1.88 ± 0.15^a,d^

XHG	21 d	35.5 ± 3.5	70 ± 6	1.87 ± 0.14
	42 d	81.9 ± 7.8	142 ± 12	1.73 ± 0.16^a,b,c^

Note: ADFI: average daily feed intake; ADG: average daily gain. Xylanase and FPHC were added from days 22 to 42. ^a^*P* < 0.05 vs. a CG group; ^b^*P* < 0.05 vs. a XG group; ^c^*P* < 0.05 vs. a HG group; ^d^*P* < 0.05 vs. a XHG group.

**Table 3:** Effect of xylanase and FPHC on the intestinal microbiota of broilers (*n* = 8).

Item		*Lactobacillus plantarum*	*Bacillus licheniformis*	*Bacillus subtilis*
CG	21 d	5.34 ± 0.39	2.68 ± 0.32	2.35 ± 1.29
	42 d	6.26 ± 0.83^b,c,d^	3.51 ± 0.57^b,c,d^	5.39 ± 0.64^b,c,d^

XG	21 d	5.05 ± 0.26	2.51 ± 0.30	2.89 ± 1.36
	42 d	7.04 ± 0.76^a,c,d^	3.00 ± 0.69^a,c,d^	5.58 ± 0.52^a,c,d^

HG	21 d	5.58 ± 0.31	2.55 ± 0.28	2.47 ± 1.15
	42 d	7.96 ± 0.75^a,b,d^	5.44 ± 0.61^a,b,d^	5.62 ± 0.49^a,b,d^

XHG	21 d	5.16 ± 0.35	2.62 ± 0.34	2.46 ± 1.21
	42 d	6.58 ± 0.87^a,b,c^	4.73 ± 0.75^a,b,c^	5.01 ± 0.32^a,b,c^

Note: From days 0 to 21, all groups received basal diets. Xylanase and FPHC were added from days 22 to 42. Bacterial numbers were represented as log_10_ cfu per gram of tissues. ^a^*P* < 0.05 vs. a CG group; ^b^*P* < 0.05 vs. a XG group; ^c^*P* < 0.05 vs. a HG group; ^d^*P* < 0.05 vs. a XHG group.

**Table 4:** Infected bacterial pathogens in broilers (cases).

Intestinal pathogens	CG	XG	HG	XHG
Coagulase-pos.				
*S. aureus*	5	3	4	1
*S. intermedius*	2	1	1	0
Coagulase-neg.				0
*S. lentus*	19	4	3	2
*S. simulans*	16	5	2	1
*S. cohnii*	10	3	2	1
*S. gallinarum*	5	3	3	2
*S. capitis*	4	2	1	0
*S. xylosus*	1	1	0	0
*S. hominis*	1	0	1	0
*S. auricularis*	1	1	1	0
*S. carnosus*	2	0	0	0
*S. caseolyticus*	2	0	0	0
*S. kloosi*	1	0	0	0
*S. epidermidis*	1	0	0	1
*S. arlettae*	1	0	1	0
*S. piscifermentans*	1	0	0	1
Gram-positive				
*Corynebacterium* sp.	3	1	1	1
*Stomatococcus* sp.	4	1	1	1
*Micrococcus sedentarius*	2	0	1	0
*Micrococcus varians*	1	0	0	0
*Micrococcus luteus*	2	0	1	0
*Streptococcus* sp.	1	0	0	0
Gram-negative				
*Escherichia coli*	6	1	2	1
*Moraxella* sp.	4	1	1	0
*Proteus mirabilis*	1			
*Acinetobacter* sp.	2	2	1	1
*Pseudomonas* sp.	6	0	2	1
*Yersinia* sp.	2	1	1	0

Note: CG: the broiler received basic diet; XG: the broiler received basic diet and 1200 IU/kg xylanase; HG: the broiler received basic diet and 0.1% polysaccharides from the fermentation extract of *Hericium caputmedusae* (FPHC, *w*/*w*); XHG: the broiler received basic diet, 1200 IU/kg of xylanase, and 0.1% FPHC (*w*/*w*). CG: basic diet; XG: basic diet + xylanase; HG: basic diet + FPHC; and XHG: basic diet + xylanase + FPHC.

## Data Availability

The data for the current study are available from the corresponding author upon reasonable request.
